# Putative role of an SLC45 H^+^/sugar cotransporter in mammalian spermatozoa

**DOI:** 10.1007/s00424-017-2024-9

**Published:** 2017-07-08

**Authors:** Olga Vitavska, Helmut Wieczorek

**Affiliations:** Department of Biology/Chemistry, Division of Animal Physiology, University of Osnabrück, 49076 Osnabrück, Germany

**Keywords:** Proton-coupled sugar transporter, SLC45A4, Spermatozoa maturation

## Abstract

In the present study, we describe the detection and analysis of a novel type of sugar transporter in mammalian spermatozoa. This transporter belongs to the SLC45 family for which two features are remarkable and distinguish it from other known families of sugar transporters. Firstly, SLC45 transporters recognise not only the monosaccharides glucose or fructose but also the disaccharide sucrose as a substrate. Secondly, the uptake of sugars is coupled to a proton gradient. Uptake experiments using radioactively labelled sucrose indicated a functional transporter of the SLC45 family in bull spermatozoa. Real-time PCR as well as Western blots demonstrated the occurrence of the SLC45 member A4 in mouse testis and sperms. Furthermore, immunocytochemical analysis of mouse tissues revealed that the signal of SLC45A4 was mainly located in the principle piece of spermatozoa. We postulate that the SLC45A4 transporter plays an important role in nutrition of spermatozoa during their maturation in the epididymis. Moreover, we suggest that knowledge about the presence of the SLC45A4 may be useful also for the methodical improvement of cryopreservation of mammalian spermatozoa.

## Introduction

For most mammalian cells, glucose is a key metabolic substance. Initially, it is taken up in the gut across the luminal membrane of enterocytes by SGLT1, a sodium-coupled glucose cotransporter of the SGLT family also known as the solute carrier family 5 (SLC5). After increase of its concentration in enterocytes, glucose is transported to the blood side by a facilitator of the GLUT (alias SLC2) family. Each of both families consists of more than 10 members, and most of them manage the transport of sugars into and out of cells. Regarding testis and sperm, there are only sparse reports on the occurrence of SGLTs, whereas the importance of various GLUTs appears to be evident [6, 25]. Except GLUT5, which is specific for fructose, all GLUTs found in the male reproductive system including sperm cells take up glucose, although most of them are able to transport also other hexoses such as fructose, mannose or galactose [18].

Recently, we established the solute carrier family 45 (SLC45) as a third mammalian sugar transporter family with unique features [4]. Firstly, SLC45 proteins are H^+^-dependent sugar cotransporters, and secondly, they transport not only the monosaccharides glucose or fructose but also the disaccharide sucrose. The latter feature was quite unusual because it evidently violated the common conception that sugars cannot be transported across membranes as disaccharides. The SLC45 family consists of only four members. SLC45 member A1 has been shown to be involved in the uptake of glucose into brain cells during hypercapnia [26], while SLC45A2 seems to play a significant role in melanin synthesis [14, 20, 29]. SLC45A3 has recently been shown by us to occur in the kidney medulla where it may transport sucrose as a compatible osmolyte from the urine into collecting duct cells [33]. Furthermore, SLC45A3 was also found in oligodendrocytes where it seems to be involved in myelin maintenance [27]. Physiological functions of the SLC45 member A4 have so far not been uncovered.

In the present study, we investigate the occurrence and putative function of SLC45 proteins in the male reproductive system, since our earlier work had localised mRNA encoding SLC45A3 and A4 in the testis in appreciable amounts [4]. We show that sucrose and glucose or fructose are transported by bull spermatozoa in an H^+^-dependent manner, and that the SLC45A4 protein is present in their plasma membrane. We discuss the possibility that SLC45A4 is especially important for sugar uptake in the epididymal lumen where the maturation of sperm cells takes place in an acidic milieu.

## Materials and methods

### Animals

Young adult male C57BL/6 mice were received from Charles River Laboratories International, kept for 1–2 weeks at standard conditions and sacrificed at the age of 12–14 weeks by cervical dislocation in accordance with the German Animal Welfare Act.

### Real-time PCR

Testes were obtained from young adult C57BL/6 mice that had been sacrificed by cervical dislocation. After removal of the *Tunica albuginea*, the tissue containing mainly seminiferous tubules was collected, frozen in liquid nitrogen and kept at −80 °C for 1–3 days until RNA preparation. RNA was isolated using TRIzol Reagent (Ambion) according to the manufacturer’s protocol. Any residual DNA traces were removed using the RNase-free DNase Set (Qiagen). Concentration and purity were verified by measuring the UV absorbance using the Implen NanoPhotometer® (Implen, Germany). The cDNA was reverse transcribed from total mRNA using the ThermoScript RT-PCR System (Invitrogen) with Oligo dT primers. Real-time PCR was done in triplicates using 0.3 μM of the following specific primers: *Slc45a1*: 5′-TCAGCTACGCCATGGAGAC-3′ (fwd) and 5′-GAGGTACATCGGTCACTCC-3′ (rev); *Slc45a2*: 5′-CTGTGCAGTATCCCTGAAG-3′ (fwd) and 5′-GACATTGTCCTCTGACTCTG-3′ (rev); *Slc45a3*: 5′-CGCTATGGCCGCCGGAG-3′ (fwd) and 5′-GGTCCCGGAAGAGGTCAG-3′ (rev); *Slc45a4*: 5′-GTCCATGCTGAAGATGCCC-3′ (fwd) and 5′-ATGACCAGGCCCCAGCAG-3′ (rev) and using Maxima SYBR Green system (Thermo Scientific) according to the manufacturer’s instructions. Ribosomal phosphoprotein P0 was used as the reference gene with the following primers: 5′-GCGACCTGGAAGTCCAACT-3′ (fwd) and 5′-GGTCCTCCTTGGTGAACAC-3′ (rev) [2]. The PCRs without template served as negative controls for sample contamination. Reactions were performed in a thermal cycler (iCycler, BioRad) according to the following protocol: at 95 °C for 10 min and 35 cycles at 95 °C for 30 s, at 58 °C for 20 s and at 72 °C for 30 s. Specificity of the primers was verified by agarose gel electrophoresis and melting curve analysis. In addition, PCR products were isolated from agarose gels and confirmed by sequencing (Seqlab, Göttingen). Mean normalised expression levels of target genes were calculated from cycle thresholds (C_T_) values as described by Simon [28].

### Isolation of spermatozoa from mouse epididymis

Caput epididymis and Cauda epididymis were isolated from young adult C57BL/6 mice and hackled slightly with a scalpel. For release of spermatozoa, tissues were incubated in a shaker for 10 min at 700 rpm and 20 °C in phosphate-buffered saline (PBS; 10 mM sodium phosphate buffer and 150 mM NaCl, pH 7.4) containing 0.3 mg/ml bovine serum albumin and a protease inhibitor mix (Roche Diagnostics). After spinning down of tissue debris at 100×*g* for 30 s, the supernatant containing spermatozoa was collected and used for further experiments. For immunocytochemistry, the spermatozoa were dropped onto SuperfrostPlus microscope slides, dried 10 min at 40 °C, fixed with methanol for 10 min and treated further as described for cryosections. For Western blot, the spermatozoa were washed twice with PBS and then heated at 95 °C for 3 min in SDS-PAGE sample buffer (0.125 M Tris pH 6.8, 2% SDS, 5% sucrose, 0.005% bromophenol blue and freshly added 2% 2-mercaptoethanol).

### Immunocytochemistry and Western blot

For detection of murine SLC45A4 protein, a peptide antibody against the sequence DGSPPFPDEVQSEHE was developed and purified using an antigen-specific column. This was done by Charles River Laboratories on our request.

Testes were isolated and fixed for 3 h at room temperature in *Lawdowsky’s* fixative (40% ethanol, 10% formaldehyde and 4% acetic acid in water). After washing with PBS, tissues were incubated gradually in 10, 20 and finally 30% sucrose/PBS at room temperature for at least 1 h, were embedded by tissue-freezing medium (Leica-Microsystems) and frozen in melting iso-pentane chilled in liquid nitrogen. Sections of 6 μm were cut on a cryostat (Leica-Microsystems) at a chamber temperature of −20 °C and collected on SuperfrostPlus microscope slides (Menzel-Gläser, Germany). Cryosections were brought to room temperature, washed with PBS and then blocked with 2% bovine serum albumin in PBS/0.1% Triton X-100 for 1 h. Incubation with the primary antibody at a dilution of 1:250 in blocking buffer was performed overnight at 4 °C. After rinsing three times for 5 min with PBS/0.1% Triton, sections were treated for 1 h at room temperature with Cy3-conjugated anti-rabbit antibody (Sigma, at a 1:250 dilution) and DAPI (4,6-diamidino-2-phenylindole, 5 μg/ml) in blocking buffer. After three washing steps of 5 min with PBS/0.1% Triton, sections were covered with Vectashield (Vector Laboratories), viewed with a confocal laser scanning microscope (Zeiss, LSM 510 Meta) and analysed using Zeiss LSM Image and ImageJ Software.

For Western blots, mouse testes lacking the *T. albuginea* were gently homogenised in cold 300 mM mannitol, 5 mM EDTA, 20 mM Tris–HCl, pH 7.5 containing a protease inhibitor mix and centrifuged 5 min at 400×*g* and 4 °C in order to remove cell nuclei and debris. Resulting supernatant was collected and divided into two portions. One of them was boiled with Laemmli buffer and served for Western blot as total cell extract. The other portion was centrifuged for 1 h at 100,000×*g* and 4 °C in order to separate cell membranes from the cytosolic fraction. Pellet was resuspended and centrifuged again under the same conditions. The resulting pellet was resuspended in homogenisation buffer, boiled with SDS-PAGE sample buffer and used for SDS-PAGE as sample for membrane fraction.

After SDS-PAGE, proteins were transferred onto a nitrocellulose membrane and probed with the SLC45A4 antibody. For this purpose, membranes were first blocked with 5% skim milk powder in TNNT buffer (20 mM Tris−HCl pH 7.5, 0.5 M NaCl, 0.02% NaN_3_, 0.05% Tween 20) for 1 h and then incubated with primary antibody at a dilution of 1: 2500. After three washing steps with TNNT buffer for 5 min each, the membranes were incubated with an anti-rabbit antibody conjugated to alkaline phosphatase (Sigma). Treatment of membranes with the primary as well as with the secondary antibody was performed in 2.5% milk/TNNT for 1 h at room temperature.

To test for unspecific binding of the primary as well as secondary antibody, control sections or control nitrocellulose membranes were probed with primary antibody saturated by a tenfold excess of the antigen peptide for 1 h at room temperature.

### Transport assays

Transport assays were performed using the spermatozoa from bull ejaculate obtained from the Osnabrueck Herdbook Cooperative. Semen samples were collected from Holstein cattle males of proven fertility by using an artificial vagina and showed more than 70% progressive motility. Transport assays were performed on the day of semen collection.

Spermatozoa isolation was performed either by centrifugation in a Percoll gradient or by the “swim-up” method [1]. One millilitre of liquefied ejaculate was loaded onto a Percoll gradient (2 ml of 80%, 2 ml of 40% Percoll in PBS) and centrifuged 20 min at 700×*g* and 20 °C in a swing out rotor. The fractions between 40 and 80% Percoll containing spermatozoa were collected. By swim-up, 1 ml of ejaculate was layered with 2 ml DMEM medium (Biochrom) and incubated 1 h at 37 °C and 5% CO_2_ in tubes that were inclined at 45 degrees. The uppermost 1.5 ml of medium was collected and centrifuged 5 min at 500×*g* for separation of spermatozoa.

Isolated spermatozoa were washed three times in uptake buffer containing (except where indicated otherwise) 40 mM potassium phosphate, 150 mM NaCl, pH 6.0 by re-suspending and centrifugation at 500×*g* for 5 min. After a pre-incubation at 37 °C for 5 min in this buffer with 50 μM carbonyl cyanide *m*-chlorophenylhydrazone (CCCP) and 0.1% DMSO or with 0.1% DMSO only, the uptake assay was started by adding ^14^C-sucrose (final concentration of 10 mM, specific radioactivity of 2.5 μCi/ml, purchased from Hartmann Analytic, Germany). Reactions were performed for 3 min (except the time dependence experiments) at 37 °C and were stopped by 20-fold dilution of the sample with ice-cold PBS. Then cells were washed twice with PBS and lysed in 0.1% sodium dodecyl sulphate. Accumulated ^14^C-sucrose was measured by scintillation counting (Beckman LS 6500).

For competition assays, competitors were added at a final concentration of 40 mM together with 10 mM ^14^C-sucrose. After 3 min of incubation, the sucrose uptake was analysed and compared to uptake rates without any competitor. All sugars were obtained from Sigma in a purity ≥99.5%.

The CCCP inhibitable part of sucrose uptake was determined in each single experiment by subtraction of uptake values in the presence of CCCP from uptake values measured without CCCP.

Statistical analysis of all transport experiments was performed by two-tailed student’s *t* test.

## Results

### Proton-dependent sugar cotransport in mammalian spermatozoa

**Fig. 1:**
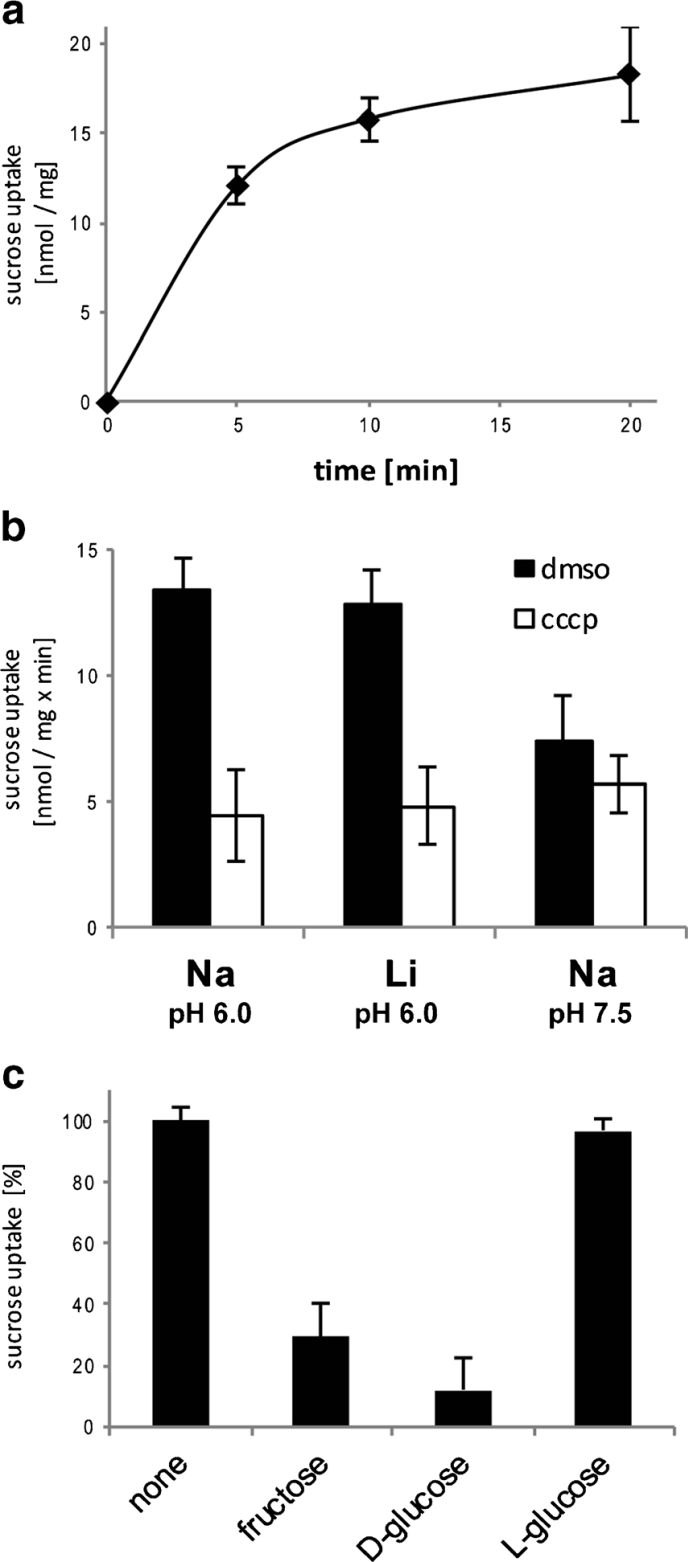
Proton-dependent sucrose uptake in bull spermatozoa. Spermatozoa were purified from fresh bull ejaculate by centrifugation in a Percoll gradient, washed three times with corresponding uptake buffer and used for uptake experiments with ^14^C-sucrose. Uptake of ^14^C-sucrose by bull spermatozoa was investigated at 37 °C in the presence of 50 μM CCCP or with DMSO as a control. **a** Time dependence. Uptake of 10 mM ^14^C–sucrose was performed in 40 mM potassium phosphate, 150 mM NaCl, pH 6.0. The CCCP inhibitable part of uptake is shown. **b** Coupling to a proton gradient. Uptake was performed for 3 min in three buffer systems as follows: Na pH 6.0: 40 mM potassium phosphate, 150 mM NaCl, pH 6.0; Li pH 6.0: 40 mM potassium phosphate, 150 mM LiCl, pH 6.0; Na pH 7.5: 40 mM potassium phosphate, 150 mM NaCl, pH 7.5. **c** Substrate specificity. Uptake was performed for 3 min in 40 mM potassium phosphate, 150 mM NaCl, pH 6.0 with 10 mM ^14^C-sucrose alone (none) or in the presence of 40 mM of one of the putative competitors. The figure presents the CCCP inhibitable part of uptake. Data points of **a**, **b** and **c** correspond to the means ± s.d. of three independent experiments

In order to check whether mammalian spermatozoa express transport proteins of the SLC45 family in their plasma membrane, we performed uptake experiments with radioactively labelled sucrose in the absence and in the presence of the specific protonophore carbonyl cyanide *m*-chlorophenylhydrazone (CCCP), which breaks down a proton gradient and is widely used in physiology to indicate a proton-coupled mechanism of transport [11, 23]. Both, transport of the disaccharide sucrose and coupling of sugar transport to a proton gradient, are remarkable features of only the SLC45 family [4]. Thus, the CCCP inhibitable part of sucrose uptake monitors sucrose transport mediated exclusively by members of the SLC45 family. For uptake assays, we used spermatozoa purified from bull ejaculate and found that they indeed accumulated ^14^C-sucrose in the presence of a proton gradient. Figure 1a presents the time course of the CCCP inhibitable part of sucrose uptake. The experiments were done in the presence of 150 mM Na^+^ in order to comply with the physiological sodium ion concentration in extracellular compartments of animal cells. In this case, a Na^+^ gradient also could support sucrose transport into the cells. To be sure that sucrose transport is coupled only to a proton but not, in addition, to a sodium motive force as it is the case for SGLTs, we measured the uptake of sucrose in a buffer containing lithium ions instead of sodium ions. Figure 1b demonstrates that Na^+^ does not have any effect on sucrose transport in spermatozoa. Thus, total sucrose accumulation (black columns) in spermatozoa at pH 6.0 was nearly the same in the presence of Na^+^ as well as in the presence of Li^+^ (*p* = 0.6). By contrast, sucrose uptake in the presence of Na^+^ decreased dramatically at pH 7.5 (*p* = 5.3E-5). The reduction of sucrose uptake at pH 7.5 compared to that at pH 6.0 provided additional evidence for a proton-dependent cotransport. Thereby, background values (white columns) of sucrose uptake in the presence of CCCP in all three buffer systems showed very similar values (*p* was between 0.21 and 0.82 at all combinations). Finally, comparison of the CCCP inhibitable part of sucrose uptake makes our conclusion even clearer. At pH 6.0, sucrose uptake in the presence of Na^+^ was similar to that in the presence of Li^+^ (*p* = 0.11), while elevation of pH from 6.0 to 7.5 led to significant decrease in transport rate (*p* = 3.1E–8). The fact that sucrose uptake was not completely deleted in the presence of CCCP is not unexpected because the sucrose gradient itself provides a part of the driving force for transport [4, 33].

In our previous studies, we have found that members of the SLC45 family are able to recognise not only the disaccharide sucrose but also the monosaccharides glucose or fructose as physiological substrates [4, 33]. To check the substrate specificity of the SLC45 transporters in spermatozoa, we performed competition assays using glucose or fructose as putative competitors. In line with our expectation, the presence of glucose or fructose reduced the proton-dependent uptake of sucrose dramatically (Fig. 1c). By contrast, L-glucose, a biologically inactive isomer of glucose, had no effect on the transport rate, thus verifying the proton-coupled uptake of sucrose in bull spermatozoa as a specific transport process. Furthermore, the fact that the addition of 40 mM L-glucose had no influence on sucrose uptake, although other competitors did, suggests that the increased osmolality had no effect on sugar transport.

### Is the SLC45A4 transporter responsible for sucrose uptake in spermatozoa?

**Fig. 2:**
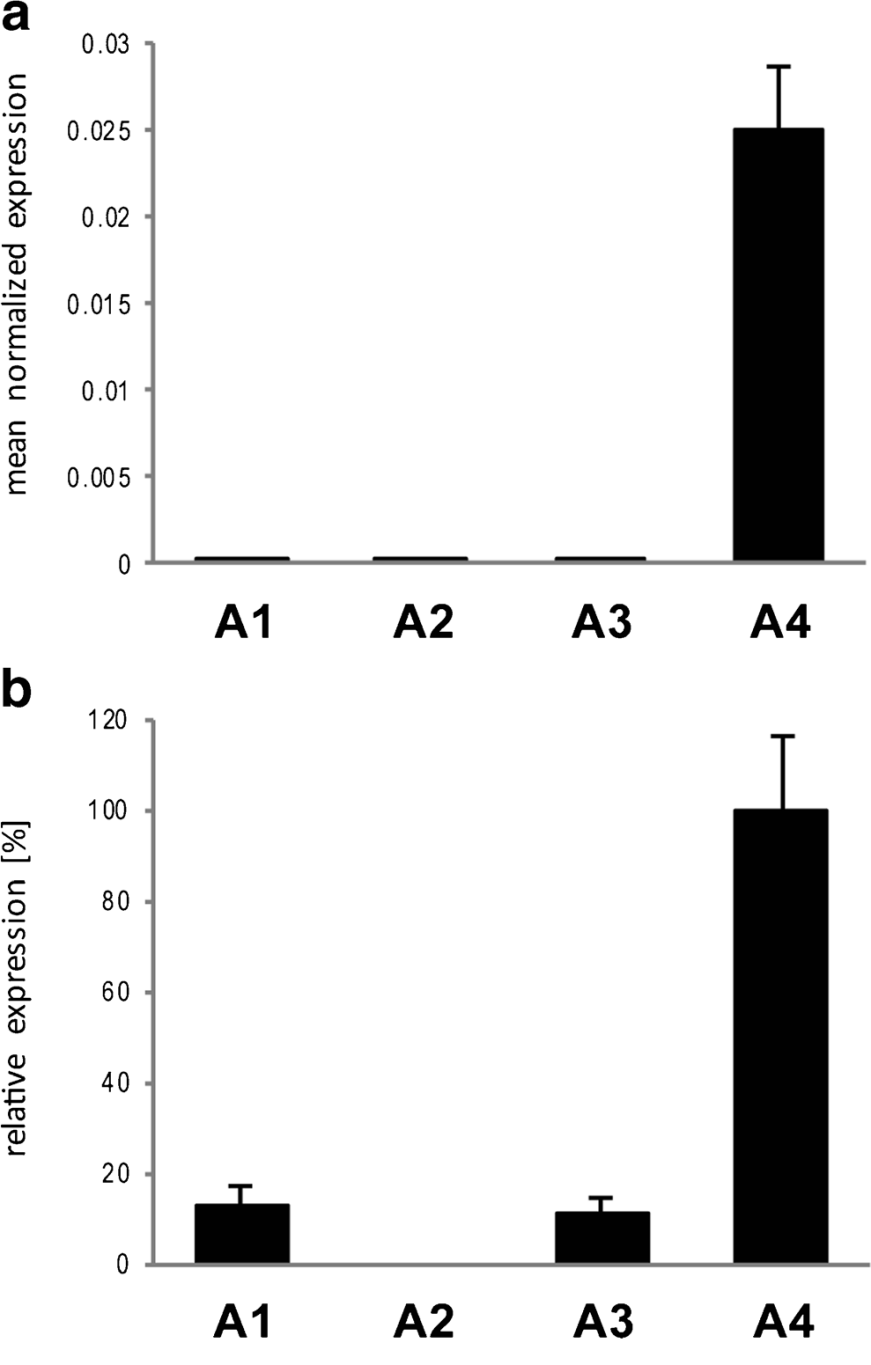
Expression of SLC45 family members in mouse testis and spermatozoa. The amount of mRNA was analysed by real-time PCR and expressed as the ratio of the sample to ribosomal phosphoprotein P0, a housekeeping gene. Data correspond to the means ± s.d. of three independent tissue preparations, and all PCRs were run in triplicates. **a** mRNA isolated from testis parenchyma. **b** mRNA isolated from spermatozoa. Normalised to housekeeping gene expression, expression of *slc45a4* gene was taken as 100% and was compared to normalised expression of *slc45a1*, *slc45a2* or *slc45a3*

The uptake assays with sucrose clearly demonstrated that at least one of the four members of the SLC45 family occurs in bull spermatozoa and was responsible for a proton-coupled cotransport of sucrose into these cells. In our studies regarding the distribution of SLC45 family members in mouse tissues [4], we previously found that the *Slc45a4* gene was predominantly and highly expressed in complete testes. However, we did not analyse the expression of the *Slc45a1* gene at that time. For our present studies, we collected parenchyma of mouse testes by removing the *T. albuginea* and analysed mRNA amounts of all four members of the SLC45 family by quantitative real-time PCR. The results shown in Fig. 2a confirmed indeed that only the SLC45 family member *a4* occurs in significant amounts in mouse testis, while *a1*, *a2* and *a3* were nearly not detectable. Because the testis tissue taken for mRNA preparation contained not only precursors of spermatozoa but also other cells types such as cells of connective tissue or Leydig cells, we analysed the expression of these genes also in mature spermatozoa isolated from mouse epididymis. Figure 2b presents the proportion between *Slc45a4* and other members of the family after normalization of their expression to the housekeeping gene and demonstrates that the *a4* member was the most prominent gene of the SLC45 family. Thus, the amount of *Slc45a4* mRNA was approximately fivefold higher than the amounts of *Slc45a1* mRNA as well as of mRNA of *Slc45a3*. The mRNA of *Slc45a2* was undetectable and comparable with the negative controls without templates.

**Fig. 3:**
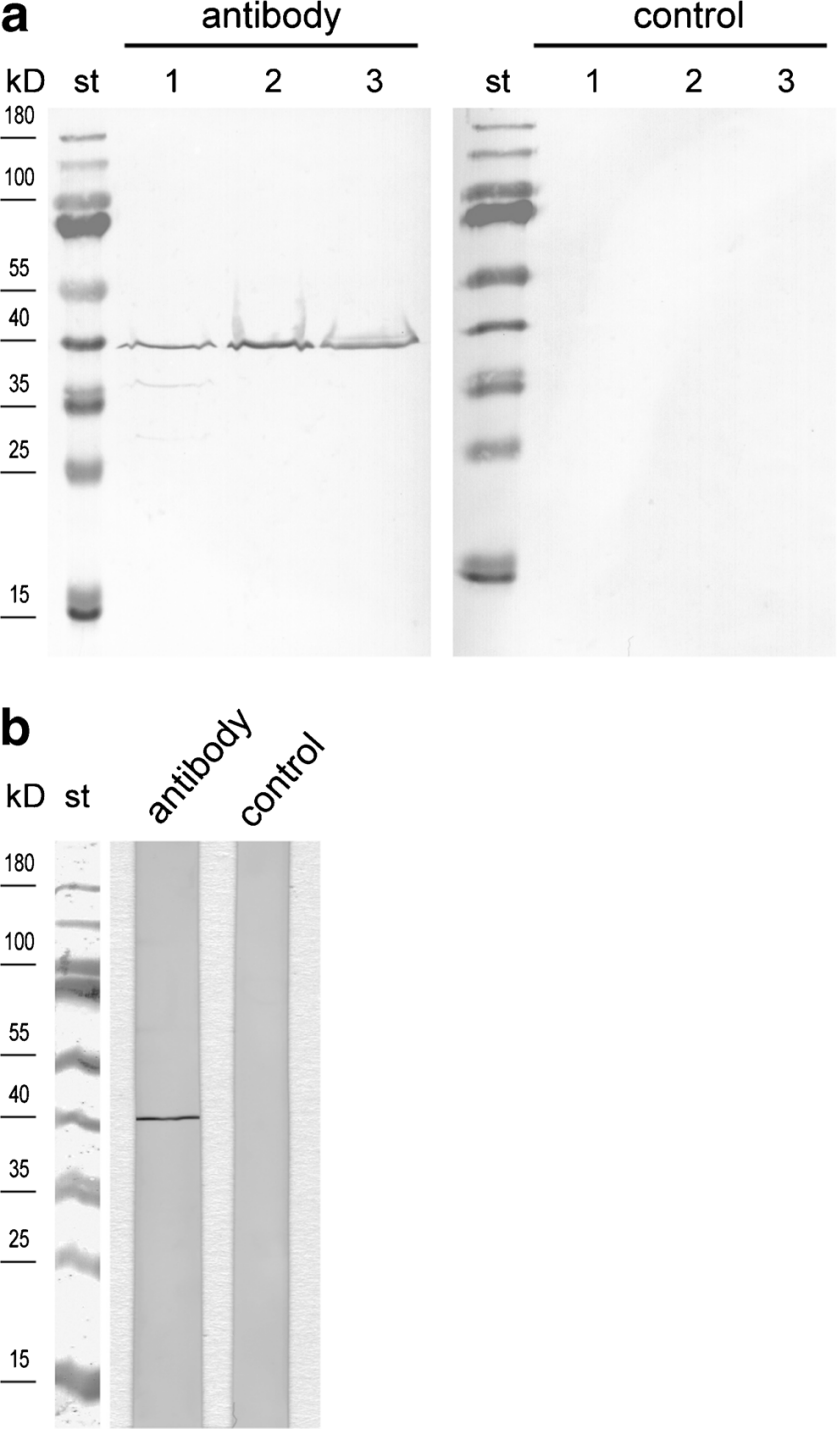
Detection of SLC45A4 in testis and spermatozoa by Western blot. Approximately 50 μg protein per gel lane was separated by SDS-PAGE, transferred onto nitrocellulose membranes and probed with an antibody against the SLC45A4 transporter. Membranes probed with antibody which had been pre-incubated for 1 h with a tenfold excess of antigen peptide were used as controls for specificity of the antibody. The primary antibody was visualised with anti-rabbit antibody conjugated to alkaline phosphatase. **a** Mouse tissues. St—Thermo Scientific PageRuler™ Prestained Protein Ladder, *1*—testis crude extract, *2*—membrane fraction of testis crude extract, *3*—crude extract of spermatozoa isolated from epididymis. **b** Crude extract of spermatozoa isolated from bull ejaculate. *St*—Thermo Scientific PageRuler™ Prestained Protein Ladder. *Figures* show representative views of three independent preparations

**Fig. 4:**
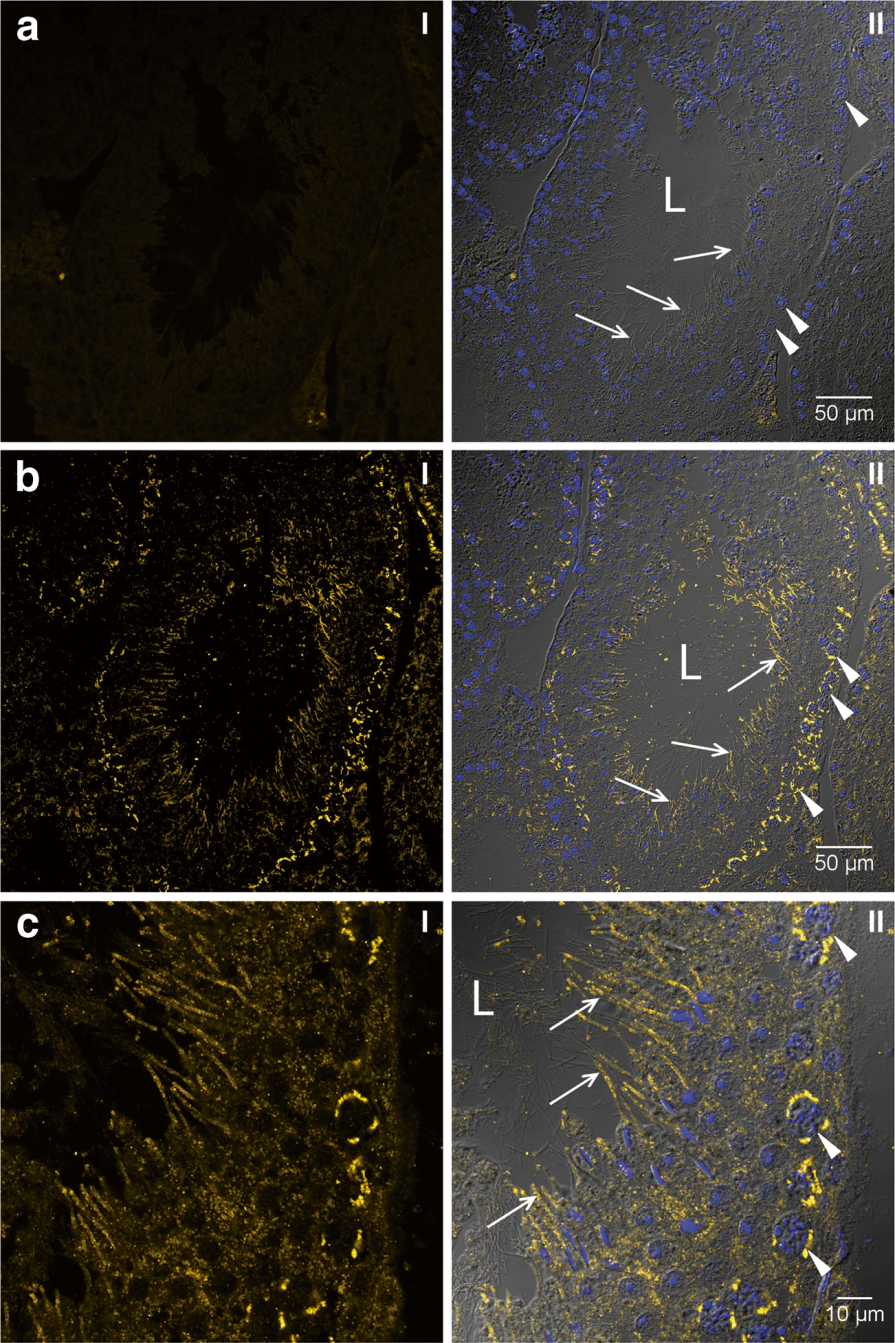
Localisation of SLC45A4 transporter in mouse testis. Six micrometres of cryosections of mouse testis were probed with an antibody against the SLC45A4 transporter. Primary antibody was visualised with anti-rabbit antibody conjugated to Cy3 (*yellow*). Cell nuclei were stained with DAPI (*blue*). Sections were analysed by a laser scanning microscope, and pictures present 1-μm optical sections. **a** Overview of a control section probed by primary antibody after its saturation with antigenic peptide. **b** Overview of an examined section. **c** Detailed view of **b**. *I* SLC45A4 signal, *II* composite of SLC45A4 signal with brightfield and DAPI staining. *Figures* show representative views of three independent experiments. Overall, roughly 170 examined and 110 control cryosections were analysed. *L* lumen of a seminiferous tubule; *arrows* point to tails of spermatids; *arrow heads* indicate spermatogonia

**Fig. 5:**
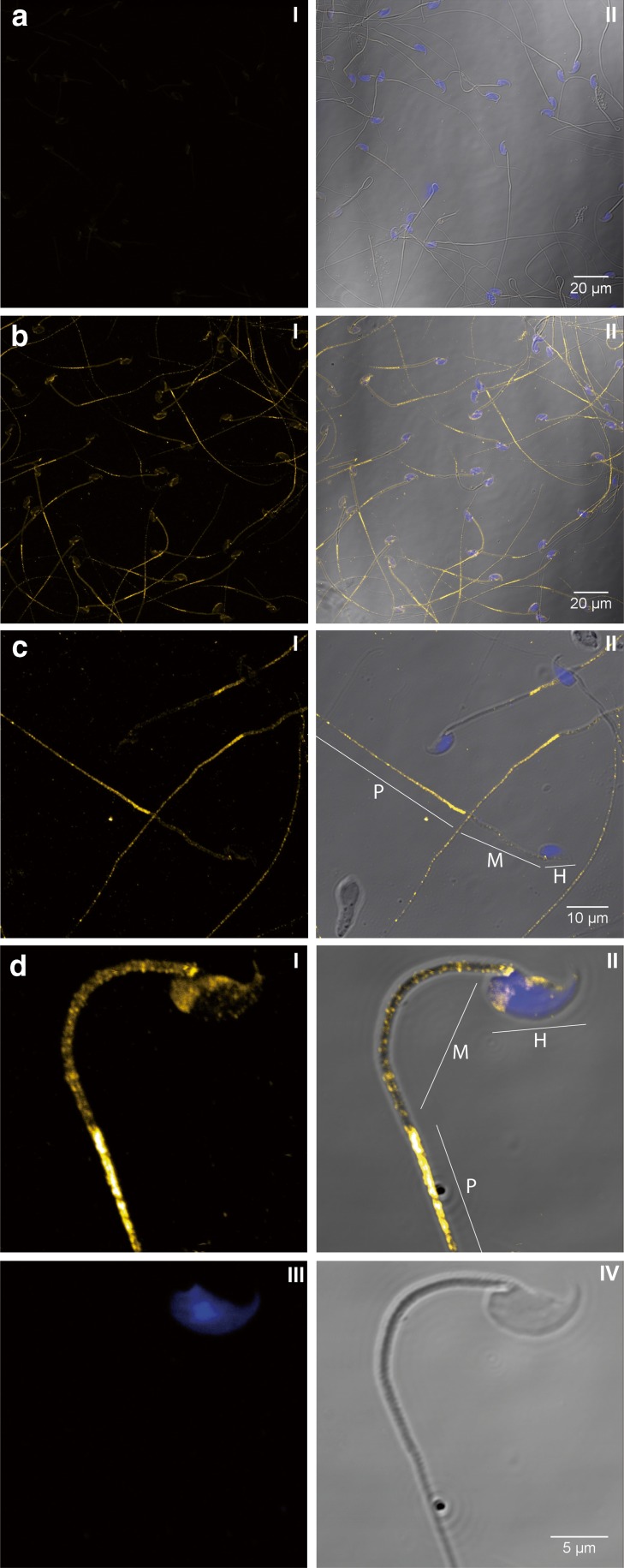
Localisation of SLC45A4 transporter in mouse spermatozoa. Spermatozoa from mouse epididymis were probed with an antibody against the SLC45A4 transporter and analysed by a laser scanning microscope. Primary antibody was visualised with anti-rabbit antibody conjugated to Cy3 (*yellow*). Cell nuclei were stained with DAPI (*blue*). **a** Overview of a control preparation probed by primary antibody after its saturation with antigenic peptide. **b** Overview of an examined preparation. **c** Detailed view of **b**. **d** Detailed view of a single sperm as a 0.5-μm optical section. *I* SLC45A4 signal, *II* composite of SLC45A4 signal with brightfield and DAPI staining; *III* DAPI staining, *IV* brightfield, *H* head, *M* midpiece, *P* principal piece of spermatozoon. *Figures* show representative views of 18 examined and 12 control samples from three independent tissues preparations

In order to localise the SLC45A4 transporter also on protein level, we developed a peptide antibody against an amino acid sequence from the central cytosolic loop of this transporter. Due to a blast search in the mouse database, this amino acid sequence occurs only in the SLC45A4 protein. In Western blots, a single band was detected not only in crude extracts from mouse testis and spermatozoa but also in the membrane fraction of mouse testis, suggesting specificity of the antibody (Fig. 3a). Furthermore, the antibody recognised a single band also in crude extract of bull spermatozoa isolated from ejaculate (Fig. 3b). We performed immunocytochemistry using this antibody in order to localise the SLC45A4 transporter in mouse testis. Control cryosections treated with antibody saturated with antigen peptide did not show any unspecific signals (Fig. 4a), thus verifying suitability of the antibody for immunocytochemistry. In examined cryosections of mouse testis, we found the SLC45A4 signal actually neither in Leydig cells nor in cells of the connective tissue but in cells of seminiferous tubules (Fig. 4b). Thereby, the signal was predominantly detected in spermatogonia and mature spermatids (Fig. 4c). In spermatogonia, the SLC45A4 signal seemed to be located at the plasma membrane, and in spermatids, the signal appeared in the tail rather than in the head region. Finally, to clear the localisation of the SLC45A4 also in spermatozoa, the sperms collected from mouse epididymis were investigated. Like in spermatids, the signal of the SLC45A4 was mainly localised in the tail of spermatozoa (Fig. 5b), while controls with saturated antibody showed no signals (Fig. 5a). Interestingly, this signal was specifically concentrated in the principal piece (Fig. 5c), while the signal in the head and in the midpiece of spermatozoa was present but sparse (Fig. 5d).

## Discussion

### SLC45 sugar transporters occur in mammalian spermatozoa

In the present paper, we describe the discovery and analysis of a novel type of sugar transporter in mammalian spermatozoa: a proton-coupled cotransporter belonging to the SLC45 family. At first, we checked the presence of the SLC45 protein by “proof of function” and found indeed a proton-dependent uptake of sucrose into bull spermatozoa isolated from the ejaculate (Fig. 1). Because the uptake of the disaccharide sucrose is, besides proton-dependent cotransport, a second feature of the SLC45 family, we used radioactively labelled sucrose as an experimental model in order to investigate only transporters of this family and to eliminate largely the effect of any other sugar transporters like SGLTs or GLUTs. The measured uptake rate of approximately 2 nmol sucrose per min and mg protein is rather low but still in the same range as H^+^/sucrose cotransport in other mammalian cells such as NRK (Normal Rat Kidney) cells [33]. Furthermore, the affinity of the transporter to glucose or to fructose appeared to be markedly higher than that to sucrose (Fig. 1b). Actually, glucose and fructose, but not sucrose, occur in fluids of the male reproductive tract and evidently are the real physiological substrates also for the SLC45 transport proteins.

For the uptake experiments, we used spermatozoa purified from bull ejaculate by centrifugation in a Percoll gradient. In this way, the spermatozoa were safely separated from other cells in the ejaculate such as epithelial cells of the reproductive tract, leukocytes or bacteria [1, 12, 39]. Although centrifugation in a Percoll gradient is a very trustworthy and convenient purification method, we also used the “swim-up” technique for preparation of spermatozoa from semen [1]. In contrast to centrifugation in a Percoll gradient, the swim-up purification allows spermatozoa the access to nutrients for most of the preparation time, but the separation from other cell types is probably not very efficient. We performed uptake experiments with spermatozoa purified by swim-up only once as a proof of principle and observed nearly the same transport rates (data not shown) as in spermatozoa purified by a Percoll gradient. This means that the results of our sucrose uptake experiments indeed reflected transport processes in intact spermatozoa.

### SLC45A4, a candidate for H^+^-coupled sugar transport in spermatozoa

The next question was to clarify which one of the four members of the SLC45 family was responsible for the sucrose uptake in spermatozoa. The results of real-time PCR as well as of Western blot analysis point at the SLC45A4 transporter. Surprisingly, the single band in Western blots was located in all cases at a molecular mass of approximately 40 kDa which is roughly half of the expected SLC45A4 molecular mass of 85 kDa. We assume some post-translational modification of the SLC45A4 protein as the most probable cause for this discrepancy, although our finding might in principle also suggest that the antibody recognises a different, unknown protein. However, the SLC45A4 protein has to occur in testis and spermatozoa because we detected the respective mRNA in both locations. Although the mRNA amount does not inevitably correlate with the respective protein amount, mRNA expression in tissues is in general a strong indication for protein occurrence, too. Thus, the SLC45A4 protein should be detectable in Western blots of testis and spermatozoa, especially because proteins are denatured after SDS-PAGE, and therefore, the antigen epitope of SLC45A4 should be accessible for the paratope of the peptide specific antibody. Binding of the antibody to a second protein with a similar sequence should most probably have led to a further band in the Western blot. Interestingly, comparable findings were also reported in the Human Atlas Project where an antibody produced against human SLC45A4 recognised bands not corresponding to the predicted size [31]. In order to understand the role of SLC45A4 in sperm development, we investigated mouse testis and spermatozoa from mouse epididymis by immunocytochemistry. In mouse testis, the signal for SLC45A4 was detected only in germ cells at every developmental stage, starting in spermatogonia. In this context, the signal in filament-like structures at the side facing the lumen, which surely represented the flagellum parts of germ cells, is especially interesting. However, we were unable to reliably differentiate between spermatozoa and their precursors, elongated spermatids. In spermatozoa isolated from the epididymis, a strong signal of SLC45A4 was found in the principal piece of spermatozoa also called tail or flagellum (Fig. 5c). In the head or in the midpiece of sperm, the SLC45A4 protein was also detected but at a lower concentration (Fig. 5d). The midpiece region is located between the head and flagellum and represents the mitochondria containing region of spermatozoa, where energy is predominantly produced by oxidative phosphorylation. Although oxidative phosphorylation is 15 times more efficient than glycolysis regarding the generation of ATP per molecule of oxidised glucose, glycolysis, however, still appears to be a canonical metabolic pathway in mammalian spermatozoa [9, 19, 30], evidently because no oxygen is needed and because glycolysis is 100 times faster than oxidative phosphorylation [34]. Glycolytic enzymes are concentrated in the principal piece of sperms [10, 36, 37] suggesting that here, glycolysis is critical for the normal function of spermatozoa [19, 30]. The spatial proximity of SLC45A4 to the place of glycolysis provides, without any doubts, a crucial advantage for energy generation. For comparison, most of the GLUTs also were found in the principal piece of spermatozoa [3, 5].

### Why do spermatozoa need a proton-coupled sugar transporter at all?

Mammalian spermatozoa face a slightly acidic milieu only in the epididymis. In the seminiferous tubules, the pH of the luminal fluid is approximately 7.3, then falls, starting from the initial region of epididymis, down to about 6.5 in the caput and achieves values of roughly 6.8 in the cauda epididymis [15, 16]. The acidification of the epididymal lumen is necessary for maturation of spermatozoa [22, 40]. Spermatozoa leaving the testis are non-functional gametes, and only during the transit through the long convoluted tubule, the epididymis, they undergo maturation necessary for progressive motility and finally for fertilization of ova. Each region of the epididymis has distinctive functions: caput and corpus are responsible for early and late sperm maturational events, respectively, while in the cauda, the functionally mature spermatozoa are stored. Interestingly, up to 40% of infertile men exhibit idiopathic infertility reflecting sperm maturational disorders [7].

Because a pH of 6.5 is optimal for the transporters of the SLC45 family [4], we propose that the SLC45A4 proton-coupled sugar transporter may play an important role in nutritional support during maturation of spermatozoa in the epididymis. We assume that the SLC45A4 transporter is physiologically important only as long as spermatozoa remain in the epididymis. Later at neutral pH in the ejaculate or in the female reproductive tract, the transporters of the SLC45 family may no more be relevant.

In general, a secondary active transporter concentrates substrates against their own electrochemical gradients and may be, in this way, much more powerful than a facilitator which transports compounds energetically only downwards. A transporter of the SLC45 family thus could support the spermatozoa with glucose or fructose more efficiently than GLUT proteins, provided a proton-gradient exists across membrane. Although the functions of the SLC45A4 protein in spermatozoa have still not been clarified ultimately, our results help to understand physiological processes in mammalian spermatozoa.

In the end, we speculate that the presence of a proton-coupled sugar transporter in sperms might also be important for the cryopreservation of these cells. Spermatozoa were the first cell type to be successfully frozen and thawed [24] and are widely used for artificial insemination in several animal species [8, 21, 32]. However, a major critical factor during cryopreservation is osmotic stress [17, 35]. During freezing, sperms are exposed to increasingly hyperosmotic conditions, because water freezes out of solution, and later by thawing, the hyperosmotic conditions decrease due to ice melting. For protection of cells during cryopreservation, a number of osmolytes, compatible as well as non-compatible, have been tested for years. Non-compatible osmolytes cannot cross the cell membrane and thus dehydrate the cells. In contrast, compatible osmolytes are, in addition, transported across the cell membrane and may protect the cells from excessive dehydration. In this context, the disaccharide sucrose was also used many a time but understandably considered as a non-compatible osmolyte (e.g. [13, 38, 41]). Since currently cryopreservation is performed at a neutral to slightly alkaline pH, sucrose indeed behaves as a non-compatible osmolyte because SLC45 family transporters are not active under this condition. A shift from neutral to a slightly acidic pH would turn sucrose into a compatible osmolyte as well. Furthermore, glucose and fructose, the key nutrients in spermatozoa, could be taken up by a proton-sugar cotransporter more efficiently than by uniporters of the GLUT family. Thus, we speculate that a change of the pH to 6.5 and the addition of sucrose may positively affect the survival of spermatozoa by cryopreservation: a slightly acidic milieu would intensify the uptake of glucose and fructose for nutritional reasons while sucrose could serve as a dual (compatible and non-compatible) osmolyte.
